# Effect of whole-body electromyostimulation and / or protein supplementation on obesity and cardiometabolic risk in older men with sarcopenic obesity: the randomized controlled FranSO trial

**DOI:** 10.1186/s12877-018-0759-6

**Published:** 2018-03-09

**Authors:** Wolfgang Kemmler, Matthias Kohl, Ellen Freiberger, Cornel Sieber, Simon von Stengel

**Affiliations:** 1 Institute of Medical Physics, FAU Erlangen-Nürnberg, Henkestrasse 91, 91052 Erlangen, Germany; 2 Faculty of Medical and Life Sciences, University of Furtwangen, Schwenningen, Germany; 3 Institute for Biomedicine of Aging, FAU Erlangen-Nürnberg, Nürnberg, Germany

**Keywords:** Sarcopenic obesity, Obesity, Protein, Exercise, Electromyostimulation, Older men

## Abstract

**Background:**

Sarcopenic Obesity (SO) is characterized by low lean and high fat mass; i.e. from a functional aspect a disproportion between engine (muscle) and mass to be moved (fat). At present, most research focuses on the engine, but the close “cross talk” between age-associated adipose and skeletal muscle tissue inflammation calls for comprehensive interventions that affect both components alike. Protein and exercise are likely candidates, however with respect to the latter, the enthusiasm for intense and frequent exercise is rather low, especially in functionally limited older people. The aim of this study was therefore to evaluate the effect of whole-body electromyostimulation (WB-EMS), a time-efficient, joint-friendly and highly customizable exercise technology, on obesity parameters and cardiometabolic risk in men with SO.

**Methods:**

One-hundred community-dwelling (cdw) Bavarian men ≥70 years with SO were randomly assigned to either (a) whey protein supplementation (WPS), (b) WB-EMS and protein supplementation (WB-EMS&P) or (c) non-intervention control (CG). Protein supplementation contributed to an intake of 1.7–1.8 g/kg/body mass/d, WB-EMS consisted of 1.5 × 20 min/week (85 Hz, 350 μs, 4 s of strain–4 s of rest) with moderate-high intensity. Using an intention to treat approach with multiple imputation, the primary study endpoint was total body fat mass (TBF), secondary endpoints were trunk fat mass (TF), waist circumference (WC) and total-cholesterol/HDL-cholesterol ratio (TC/HDL-C).

**Results:**

After 16 weeks of intervention, TBF was reduced significantly in the WPS (− 3.6 ± 7.2%; *p* = 0.005) and WB-EMS&P (− 6.7 ± 6.2%; *p* < 0.001), but not in the CG (+ 1.6 ± 7.1%; *p* = 0.191). Changes in the WB-EMS&P (p < 0.001) and the WPS group (*p* = 0.011) differ significantly from the CG. TF decreased in the WB-EMS&P (*p* < 0.001) and WPS (*p* = .117) and increased in the CG (*p* = .159); WC decreased significantly in the treatment groups and was maintained in the CG. Lastly, the TC/HDL-C ratio improved significantly in the WB-EMS&P and WPS group and was maintained in the CG. Significant differences between WB-EMS&P and WPS were determined for waist circumference only (*p* = 0.015; TBF: *p* = 0.073; TF: *p* = 0.087; TC/HDL-C: *p* = .773).

**Conclusion:**

Moderate-high dosed whey protein supplementation, especially when combined with WB-EMS, may be a feasible choice to address obesity and cardiometabolic risk in older cdw men with SO unable or unmotivated to exercise conventionally.

**Trial registration number:**

ClinicalTrials.gov NCT02857660; registration date: 05/01/2017.

## Background

Sarcopenic obesity (SO) is characterized by the redistribution of muscle and fat mass at increased age [1]. Most researchers focus on the functional aspect of this “geriatric syndrome” [2], however, its severe cardiometabolic implications [3] - highly relevant for morbidity and mortality of older people [1, 4] - are undisputed. Although there is an ongoing debate as to which of the two parameters, sarcopenia or obesity, dominates the molecular process related to the pro-inflammatory status of SO [5], i.e. which is the cause and which is the effect [5], the optimum therapies for both conditions are still exercise and nutrition [6]. With respect to the latter, a considerable amount of research (review in [7–12] concentrates on an optimum protein and amino acid intake in the elderly, predominately under the aspect of maintenance of muscle mass (and function). In contrast, to our best knowledge no study has determined the isolated effect of protein/amino acids on obesity and cardiometabolic risk factors in people with prevalent SO. Nevertheless, a beneficial effect of (whey) protein [13] on total and abdominal fat reduction and (other) cardiometabolic risk factors [13, 14] in overweight and obese people has been previously reported. In parallel, apart from its beneficial effect on sarcopenia, resistance exercise training (RT) favorably affects obesity and cardiometabolic risk factors in middle-aged and older adults [15–18]. However, it is unrealistic to assume that people with SO will achieve the intensity and frequency of exercise recommended for positively impacting disabling conditions or obesity [19]. In this context, whole-body electromyostimulation (WB-EMS), an effective, time-efficient, joint-friendly and highly customized further development of the recognized local EMS application predominately applied in therapy [16, 20–27], may be a good choice for older subjects at risk for sarcopenic obesity (SO).

The aim of this contribution was to determine the effect of isolated whey protein supplementation (WPS) and a combined WB-EMS and whey protein protocol on SO under particular consideration of the obesity and cardiometabolic aspect of SO in community dwelling men 70 years+ and older with SO.

Our primary hypothesis was that WB-EMS&P *but not* isolated WPS significantly affected obesity, compared with a non-training, non-protein-supplemented control.

Our secondary hypothesis was that WB-EMS&P *but not* isolated WPS significantly affected cardiometabolic risk factors compared with a non-training, non-protein-supplemented control.

## Methods

The Franconian Sarcopenic Obesity (FranSO) study is a randomized controlled trial in a parallel groups design with three balanced study arms: (a) WB-EMS and protein supplementation (b) protein supplementation (c) non-intervention control. The present contribution focuses on the “obesity” aspect of the study and (related) cardiometabolic risk factors. The corresponding “sarcopenia” aspect was specifically addressed in a recently published article [21].

The Institute of Medical Physics (IMP), Friedrich-Alexander University Erlangen-Nürnberg (FAU) conducted the study between February and December 2016. FranSO was approved by the ethics committee of the FAU (Ethikantrag 67_15b)^1^ and fully complied with the Helsinki Declaration “Ethical Principles for Medical Research Involving Human Subjects“. All study participants gave their written informed consent. The FranSO-study was fully registered under ClinicalTrials.gov: NCT2857660.

### Participants

**Fig. 1:**
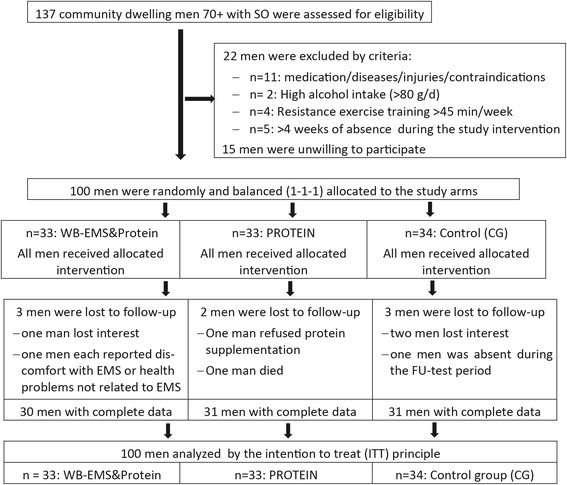
Diagram of participant flow through the different study phases

After extensive screening [28], 100 community dwelling, Caucasian males 70 years and older with the eligibility criteria listed below were randomly allocated to the three study arms (Fig. 1). Inclusion criteria apart from sex, age and status (see above) were (a) skeletal muscle mass index (SMI) < 0.789^2^ as suggested by the Foundation for the National Institutes of Health (FNIH) [29] for the sarcopenia aspect of SO. (b) A percentage body fat ratio of > 27% (PBF) representing obesity, as recommended by Baumgartner [30]. Exclusion criteria were (a) present medication or diseases affecting body composition or preventing WB-EMS application (e.g. cardiac pacemaker), (b) any type of RT conducted for > 45 min/week, (c) absence during the intervention period, (d) regular alcohol consumption > 80 g/d on 5 days/week or (e) unwillingness to accept the randomization procedure. Fig. 1 shows participant flow through the study.

### Intervention

#### Whole-body Electromyostimulation (WB-EMS)

Although WB-EMS has been frequently described (e.g. [20, 22, 23, 25, 26, 31–34]), a brief introduction of this predominately resistance type exercise will be given. WB-EMS is based on the recognized local electromyostimulation predominately applied in the treatment of muscular injuries. The main benefit of EMS might be that it can be applied independent of voluntary muscle activation up to supra-maximum level. A drawback of EMS frequently addressed is the restricted impact on functional parameters, at least when applied in a static mode (review in [35]). Unlike local EMS, current WB-EMS equipment enables the simultaneous activation of up to 10 regions or 14 muscle groups. The total stimulated area, up to 2600–2800 cm^2,^ can be simultaneously activated, with selectable intensity for each region. In the present study, we applied our video-guided WB-EMS standard protocol (bipolar, 85 Hz, impulse-width: 350 μs) in a standing position 1.5 × 20 min^3^ per week for 16 weeks. We used an interval approach with 4 s of EMS stimulation with an immediate impulse burst and an impulse pause of 4 s. During the impulse phases, different low intensity exercises were conducted [25, 26] in order to overcome the frequently reported limited impact of static EMS application on neuromuscular coordination and functional parameters. In detail, we conducted two sets of 8 different movements (Table. 1) with 6–12 repetitions. However, intensity and amplitude of the exercises/movements given in Table 1 were kept very low to ensure that the effect of the voluntary exercise per se did no impact our primary and secondary outcomes (Table. 1).

**Table 1:** “Core movements” performed under WB-EMS Application

Exercises	
1. Leg flexions (4 s) with arm-extension / leg extension (4 s) with arm-flexion	
2. Leg flexions (4 s) with trunk flexion (crunches)	
3. Leg flexions (4 s) with lat-pulleys / leg extension (4 s) with military press	
4. Standing crunch with butterfly / standing reverse fly (4 s)	
5. Leg flexions (4 s) und vertical chest press / leg extensi. (4 s) und vertical rowing	

Core exercises were also combined and/or slightly modified (i.e. twisted crunch) to generate 12 exercises that were used alternately during WB-EMS application

After 4 weeks of conditioning, we progressively increased the duration of the session from 14 min up to 20 min. Of importance, the WB-application was applied in a highly customized personal training setting with one instructor responsible for two participants. This procedure is crucial for the proper regulation of (impulse) intensity, because we have to use a rate of perceived exertion (RPE) to generate a sufficient but tolerable intensity of the EMS application. Indeed, due to the variations in current sensitivity in the various muscle groups and differences in pain sensation of the individuals an objective prescription of (impulse) intensity is not adequate. Thus, we prescribed an RPE of “6–7” (i.e. “hard+” to “very hard”) on the Borg CR10 Scale. We individually adapted this intensity during the second session and after 4, 8 and 12 weeks for each body region in close interaction between participant and instructor. Instructors started the WB-EMS application with this saved initial setting and increased the (impulse) intensity slightly every 3 min to achieve the prescribed RPE during the WB-EMS application. This procedure was conducted in very close cooperation with the participants in order to generate optimum exercise intensity.

#### Protein supplementation

Dependent on their habitual dietary intake, we gave participants of the WPS and WB-EMS&P group with whey protein powder supplements (Inkospor Active, INKO, Roth, Germany) in order to realize a total daily protein amount of 1.7–1.8 g/kg/bodymass. The supplement contained 2.8% of fat, 6.4% of carbohydrates CHO) and 80% of (whey) protein. Including a high amino acid (57%), and L-Leucine (9%) component, the chemical score of this product is 159.^4^ The protein powder was ingested with water; doses larger than 40 g were split into two lower quantities. We did not prescribe intake at a specific time of the day. All the participants were carefully instructed on how to apply the protein supplementation. They were also contacted every second week and interviewed about their proper protein (and Vitamin D) supplementation; in parallel all participants were asked to maintain their dietary habits including dietary protein intake during the intervention.

#### Vitamin D supplementation

We provided participants of all study arms with cholecalciferol (Taxofit, Cologne, Germany). Subjects were instructed to take a dose of 800 IU/d.

### Outcomes

#### Primary study outcome

- Changes in total body fat mass (TBF) from baseline to 16 week follow-up

#### Secondary study outcome

- Changes in trunk fat mass (TF) from baseline to 16 week follow-up
- Changes in waist circumference from baseline to 16 week follow-up
- Changes in total cholesterol/HDL-Cholesterol ratio from baseline to 16 week follow-up
- Changes in triglycerides (TAG) from baseline to 16 week follow-up

#### Experimental study outcome

- Changes in total visceral fat area (VFA) from baseline to 16 week follow-up

### Assessment

All tests were performed by the same method and researcher at a similar time of day (±1 h) immediately before and after 16 weeks of intervention. Great emphasis was placed on the standardization of the tests including consistent verbal test prescription. Participants were requested to avoid severe physical activity and refrain from food/beverages 24 h and 3 h respectively prior to the assessment.

#### Anthropometric data

All parameters were determined with calibrated devices. Waist circumference was determined as the minimum circumference between the distal end of the rib cage and the top of the iliac crest along the midaxillary line. Body height was assessed using a Harpender stadiometer (Crosswell, Crymych, UK), body mass and composition were determined via direct-segmental, multi-frequency Bio-Impedance Analysis (DSM-BIA, InBody 770, Seoul, Korea). Using a tetrapolar eight-point tactile electrode system that applies six frequencies (1, 5, 50, 250, 500 and 1000 kHz), this device enables the body composition of the trunk, arms and legs to be determined separately. Appendicular skeletal muscle mass (ASMM) was calculated adding the mass for upper and lower limbs. Following the sarcopenia definition of the FNIH [29], we calculated skeletal muscle mass index (SMI) as ASMM/BMI. Reliability of the DSM-BIA device to determine TBF was checked by a test-retest protocol in two studies with 2 × 25 male participants 30–50 [36] and 70+ years old [21]. Whilst refraining from food, beverages and physical activity participants were assessed twice within one hour. Resulting ICC was 0.89 (95%-CI: .88–.93) and 0.88 (95%-CI: .85–.91) in the cohort 70 + .

#### Blood sampling

Blood was drawn on a different date three days before the main assessment. After an overnight fast, blood was consistently sampled between 7:00 and 9:00 in the morning in a sitting position from an antecubital vein. Serum samples were centrifuged for 20 min at 3000 RPM and analyzed by the “Zentrallabor” of the Medical Department, FAU. Glucose, total cholesterol, HDL and LDL cholesterol and triglycerides (Olympus Diagnostica GmbH, Hamburg, Germany) were determined.

#### Questionnaires

To adequately assess baseline characteristics of the participants, a questionnaire asked for various aspects including (a) demographic parameters, (b) diseases, (c) medication, (d) operations (e) physical limitations, low-traumatic fractures, injuries or falls within the last year, (f) pain frequency and intensity at different regions (f) lifestyle, including physical activity, exercise [37] and (g) nutrition. The abridged version of the Late Life Function and Disability Instrument (LLFDI) was used to determine the self-rated physical performance of the participants [38]. After 16 weeks of intervention, all participants conducted a comparable questionnaire in order to detect changes that may affect our study endpoints. All questionnaires were carefully checked for completeness and accuracy in close cooperation between research assistants and participants.

#### Dietary protocols

In order to determine habitual dietary intake, 4-day dietary protocols were conducted by all the participants immediately before and after the trial. Participants were carefully briefed and instructed on how to keep the protocols. The consumed food was analyzed by a certified nutritionist using the Freiburger Nutrition Protocol (nutri-science, Hausach, Germany). Doubtful results, e.g. energy consumption below 1000 or higher than 3500 kcal/d, were checked together with the participant. In all cases, the men provided a second dietary protocol that was based on days more representative for their usual nutrition. Compliance with the protein and/or vitamin-D supplementation was monitored by the same nutritionist biweekly (see above).

### Changes in trial outcomes after trial commencement

No changes in trial outcomes were made after trial commencement.

### Obesity definition

We diagnosed obesity using the body-fat rate (i.e. percent body fat; TBF%) as determined by the DSM-BIA (DSM-BIA, InBody 770, Seoul, Korea) assessment and applying a cut-off point of > 27% body fat (PBF) according to the suggestion of Baumgartner [30] for SO.

### Sample size calculation

The sample size calculation of the FranSO study was based on the Sarcopenia aspect of the project.^5^ With respect to the present primary hypothesis that addresses “total body fat” (TBF), a recent study with men 65+ [26] reported a mean difference between WB-EMS (− 1.3 ± 0.9 kg) and control condition (− 0.3 ± 0.9 kg) of 1.0 ± 1.0 kg. Applying a similar, slightly more conservative approach with a mean difference of 1.0 ± 1.25 kg the sample size of 33 participants per group generates a 90% power to detect differences of TBF between the WB-EMS&P and the CG (α = .05; t-test based sample size calculation).

### Randomization procedures

One hundred participants were randomly assigned to three study arms (WB-EMS&P vs. WPS vs. CG) using strata of 5 years and a uniform allocation rate of 1:1:1 (Fig. 1). For the group allocation, lots enclosed in opaque plastic shells (“kinder egg”, Ferrero, Italy) were drawn from a bowl by the participants themselves, albeit under supervision of the primary investigator responsible for the randomization procedure (WK). Neither participants nor researchers knew the allocation beforehand. After the group allocation, WK enrolled participants and carefully instructed them about dos and don’ts.

### Blinding

The blinding strategy refer to the assessments of the study outcomes. Research assistants/outcome assessors did not know the group allocation of the participants (WB-EMS&P, WPS or CG) and were requested not to ask either.

### Biometry and statistical analysis

All participants who were randomly allocated to the three study arms at baseline were included in the intention to treat (ITT) analysis. ITT analysis and imputation of missing (follow-up) data were conducted using R statistics software with multiple imputation performed by the Amelia II program [39]. Imputation was repeated 100 times. Multiple imputation worked well in all cases. We used graphical (QQ- and box-plots) and statistical (Shapiro-Wilkes-Test) tests to validate the normal distribution of the outcomes presented here. Based on a normal distribution of the data we applied paired-samples t-test to compute intra-group changes. Differences between the groups were determined using one-way analysis of variance (*ANOVA*). The approach of Allison [40] was used to combine the outcomes of the imputation. Relevant differences in analysis of variance results (i.e. *p* < .100) were further addressed by pairwise t-test comparisons for multiple imputation with pooled standard deviation. We adjusted “p”-values for multiple testing by the method suggested by Holm [41]. Tests were all applied 2-sided. The accepted level of significance was 5%.

## Results

Baseline characteristics of the FranSO participants listed in Table 2 did not differ relevantly between the study arms. Apart from the still non-significant between group differences for baseline protein intake, baseline data reported in Table 2 is very homogeneous between the groups. Apart from the eligibility criteria-induced low SMI and high PBF, most of the parameters were representative for cdw German men 70 years+ [42, 43]. This includes measures of functional sarcopenia that had been expected to be less favorable in this cohort.

**Table 2:** Characteristics of the participants of the FranSO study at baseline

Variable	EMS&P (*n* = 33) MV (95%-CI)	WPS (*n* = 33) MV (95%-CI)	CG (*n* = 34) MV (95%-CI)	*P*-value
Age [years]	77.1 (75.6 to 78.7)	78.1 (76.3 to 80.0)	76.9 (75.2 to 78.7)	.571
Body mass index [kg]	26.2 (25.3 to 27.0)	26.3 (25.4 to 27.1)	26.0 (25.2 to 26.9)	.941
Lean body mass [kg/m^2^] ^a^	51.8 (49.9 to 53.6)	52.1 (50.3 to 54.1)	52.6 (50.5 to 54.8)	.805
Total body fat [%] ^a^	31.6 (30.2 to 32.8)	31.4 (30.0 to 32.5)	31.4 (30.1 to 32.7)	.967
Gait velocity [m/s]	1.26 (1.19 to 1.33)	1.24 (1.18 to 1.30)	1.27 (1.21 to 1.33)	.857
Handgrip-strength [kg]	33.8 (31.0 to 36.6)	33.3 (31.2 to 35.4)	34.4 (32.1 to 36.6)	.814
Number of diseases [n] ^b^	2.71 (2.38 to 3.05)	2.78 (2.36 to 3.16)	2.56 (2.16 to 2.96)	.584
Number of medications [n]	3.31 (2.80 to 3.83)	3.49 (3.02 to 3.95)	3.40 (2.91 to 3.91)	.801
LLFDI [Index]^c^	1.52 (1.32 to 1.71)	1.58 (1.39 to 1.76)	1.53 (1.35 to 1.70)	.193
Physical activity [Index]^d^	4.35 (3.84 to 4.87)	4.16 (3.65 to 4.66)	4.68 (4.10 to 5.25)	.371
Training volume [min/week]	36 (24 to 48)	35 (24 to 46)	40 (28 to 51)	.810
Energy intake [kJ/d]^e^	8967 (8385 to 9590)	8670 (7823 to 9521)	9516 (8512 to 10,488)	. 352
Protein intake [g/kg/d] ^e^	1.17 (1.06 to 1.29)	1.01 (0.90 to 1.13)	1.21 (1.06 to 1.37)	.066
Total cholesterol [mg/dl]	215 (199 to 231)	205 (194 to 217)	201 (182 to 220)	.422
HDL-cholesterol [mg/dl]	53.3 (49.7 to 56.9)	52.3 (48.4 to 56.2)	54.0 (48.5 to 59.6)	.874
LDL-Cholesterol [mg/dl]	146 (132 to 159)	137 (127 to 147)	133 (119 to 147)	.320

^a^BIA (InBody 770, Seoul, Korea)

^b^classification suggested by Schäfer [78]

^c^LLFDI: abbreviated version of the “Late Life Function Disability Instrument” [38]; 5-scaled: (1) indicate “no problem”, (5) specify “impossible to do”

^d^7-scaled (1) indicate “very low”, (7) specified “very high” [37]

^e^4-day dietary diary; 95% CI: 95% confidence interval

Participant flow through the study was shown in Fig. 1. Altogether eight subjects were lost to follow-up; dropout rates per group averaged < 10%. We observed an overall attendance rate for the WB-EMS sessions of 91 ± 7% (i.e. ≈22 of 24 sessions). Adherence to the WB-EMS protocol was determined using the subjects’ average RPEs that were recorded by the instructors after 5, 10, 15 and 20 min. RPE averaged 6.8 ± 0.3 (7 = “very hard”) with no relevant changes after the 4th week. The proper intake of the recommended dose of protein powder was assessed by checking our protein supplementation lists and records and by a follow-up compliance questionnaire. In summary, both treatment groups took a lower dose of protein powder than prescribed (WB-EMS&P: -4.2 ± 5.2 g/d; *p* = 0.119; WPS: -7.3 ± 4.8 g/d, *p* = 0.001). This was completely compensated by the increased dietary protein intake in both groups, however. Thus, total protein intake (1.78 ± 0.09 g/kg body mass/d) did not differ during the intervention period and was at the upper end of our intended total intake. In detail, all but two participants of the WB-EMS&P group (1.59 and 1.65 g/kg/d) achieved the prescribed total protein dose of 1.7–1.8 g/kg body mass/d. One participant quit the study due to uneasiness during WB-EMS application; another participant cited antipathy to consume high (whey) protein doses for its dropout. No further adverse effects were reported by the participants.

### Study outcomes

Baseline results and changes for total body fat (TBF) was listed in Table 3. Based on similar baseline values (*p* = .995), TBF was reduced significantly by 3.6 ± 7.2% in the WPS (*p* = 0.005) and 6.7 ± 6.2% in the combined WB-EMS and whey protein supplement group (*p* < 0.001). A non-significant increase of 1.6 ± 7.1% (*p* = 0.191) was determined in the control group that differed significantly from the WB-EMS&P (p < 0.001) and the WPS group (*p* = 0.011). No significant differences were observed between both intervention groups (*p* = 0.073). Correspondingly, we reject our main hypothesis that only the combined treatment group but not the WPS group significantly reduced TBF compared with a control group without protein supplementation or WB-EMS application.

**Table 3:** Total body fat changes in the three study arms

	WB-EMS&P (*n* = 33) MV (95% CI)	WPS (*n* = 33) MV (95% CI)	CG (*n* = 34) MV (95% CI)	*P*- value
Total body fat (TBF) [kg]				
Baseline	24.1 (22.3 to 25.9)	24.0 (22.2 to 25.9)	24.2 (22.2 to 26.1)	.995
Changes	−1.62 (−1.02 to −2.22)^*^	−0.87 (−.27 to − 1.47)^*^	0.39 (.98 to −.20) ^n.s.^	<.001

MV: mean value, SD: standard deviation, CI: confidence interval. *: *p* < 0.05; ^n.s.^: non-significant; exact *p*-values and pairwise comparisons are given in the text

The results for the secondary study endpoints are listed in Table 4. Again, no significant group differences were observed at baseline (*p* ≥ 0.407).

**Table 4:** Baseline values and changes of secondary study outcomes in the study groups

	WB-EMS&P (*n* = 33) MV (95% CI)	WPS (*n* = 33) MV (95% CI)	CG (*n* = 34) MV (95% CI)	P-value
Trunk body fat [kg]				
Baseline	12.5 (11.6 to 13.4)	12.6 (11.6 to 13.7)	12.7 (11.6 to 13.8)	.957
Changes	−.69 (−.35 to −.99)^*^	−.26 (.12 to −.58) ^n.s.^	0.23 (.54 to −.09) ^n.s.^	<.001
Waist circumference [cm]				
Baseline	98.3 (96.1 to 100.4)	99.7 (97.0 to 102.9)	100.2 (97.8 to .102.6)	.462
Changes	−1.94 (− 1.44 to −2.44)^*^	−0.91 (−.42 to −.1.40)^*^	−0.10 (.46 to −.67) ^n.s.^	<.001
Total cholesterol/HDL-C ratio [Index]				
Baseline	4.13 (3.76 to 4.50)	4.04 (3.75 to 4.32)	3.83 (3.50 to 4.16)	.627
Changes	−0.31 (−.15 to −.47)^*^	− 0.34 (−.21 to −.47) ^*^	−0.07 (.08 to −.22) ^n.s.^	.020
Triglycerides [mg/dl]				
Baseline	138 (116 to 160)	140 (120 to 160)	126 (99 to 152)	.627
Changes	−4.7 (−19.5 to 10.1) ^n.s^	− 4.0 (− 18.8 to 10.7) ^n.s.^	3.6 (18.5 to − 11.4) ^n.s.^	.685

MV: mean value, SD: standard deviation, CI: confidence interval. *: *p* < 0.05; ^n.s.^: non-significant; exact p-values and pairwise comparisons are given in the text

Trunk body fat decreased significantly in the WB-EMS&P (p < 0.001) and non-significantly in the WPS group (*p* = 0.117), and increased non-significantly in the CG (*p* = 0.159). Significant group differences were determined between WB-EMS&P and the CG only (p < 0.001; WPS vs. CG: *p* = 0.068; WB-EMS&P vs. WPS: *p* = 0.087). In parallel, based on comparable baseline data (WB-EMS&P: 119 ± 34 vs. WPS: 117 ± 30 vs. CG: 121 ± 33 cm^2^), “total visceral fat area” (VFA) did not change in the CG (0.4 ± 7.7%, *p* = .614) and decreased significantly in the WPS (− 3.1 ± 8.4%; *p* = .017) and WB-EMS&P groups (− 4.6 ± 8.7%; *p* = .002). Again, a significant difference was determined between the WB-EMS&P and the control group (*p* = .034).

Waist circumference decreased significantly (*p* ≤ 0.001) in both treatment groups, and was maintained in the CG (*p* = .714). Significant group differences were observed between the treatment groups and the CG (WB-EMS&P: *p* = 0.001; WPS: *p* = 0.033) and between the WB-EMS&P and WPS group (*p* = 0.015).

The ratio of total cholesterol to HDL-cholesterol changed favorably in both treatment groups (*p* < .001) and was maintained in the CG (*p* = .365). Both treatment groups differ significantly from the CG (*p* = .039 and *p* = .020) with no relevant differences between WB-EMS&P and WPS (*p* = 0.773). Looking behind the covariates, corresponding intragroup changes and group differences were predominately based on significant reductions of total cholesterol in the treatment groups (WB-EMS&P:-8.7 ± 18.9, *p* ≤ .009; WPS: -10.2 ± 17.7 mg/dl) and a non-significant increase in the CG (3.8 ± 16.0 mg/dl, *p* = .220). HDL-C levels increased in all groups (1.7 to 2.0 mg/dl). However, changes were significant (*p* = 0.046 and *p* = 0.048) in the CG and WPS group only (WB-EMS&P: *p* = .072).

In parallel, more favorable effects in both treatment groups vs control, albeit without reaching significance (*p* > 0.10 to 0.15), were observed for the ratio of LDL-C to HDL-C. In contrast, triglycerides were not significantly affected by WB-EMS application or protein supplementation. Further, no group differences were observed (Table. 4). The same is true for resting glucose, with baseline values predominately in the normal range and intragroup changes far from being significant (*p* > 0.40).

Thus, in summary we had to reject hypothesis 2 that WB-EMS&P *but not* isolated protein supplementation will significantly affect cardiometabolic risk factors compared with a non-training, non-protein-supplemented control.

### Confounders

Follow-up questionnaires and structured interviews in close interaction with the participants did not indicate relevant changes of lifestyle, diseases and medication during the study period. In parallel, none of the participants reported operations or injuries for longer than one week.

Energy intake, CHO, fat and alcohol intake as assessed by 4-day dietary protocols did not change significantly within (*p* ≥ .270) or vary between the study groups (*p* ≥ 0.606). However, as mentioned above, dietary protein intake increased significantly in the WPS (9 ± 12 g/d, *p* = .001), increased slightly in the WB-EMS&P (3 ± 16 g/d; *p* = 0.316) and was maintained in the CG (− 1 ± 16 g/d; *p* = 0.671). Corresponding group differences were non-significant (*p* ≥ 0.056).

## Discussion

The key result of the study was that both WB-EMS&Protein and isolated WPS significantly affect total and abdominal obesity and other important cardiometabolic risk factors in community dwelling men 70+ with Sarcopenic Obesity. This result could not have been necessarily expected. The FORMOsA study [22, 44], which determined the effect WB-EMS&P in cdw women 70+ with SO, generally confirmed the favorable positive effects on cardiometabolic risk factors; FORMOsA failed to generate any significant reduction of total and abdominal body fat, however. Two main reasons may contribute to this result: (a) the low frequency and intensity of the WB-EMS application and (2) the low-moderate dose of WPS (0.3 g/kg/d) supplied in FORMOsA. Thus, in order to generate a more striking effect, we applied a more challenging WB-EMS protocol and provided higher doses of whey protein. The present results confirmed this strategy. Summarizing the favorable effects on body fat and cardiometabolic risk, FranSO ranged in the upper region of combined RT/protein supplement approaches in older adults (e.g. ([45–50]).

In contrast to the recognized effect of combined resistance type & protein approaches, the clinical effectiveness of isolated protein and/or amino acid supplementation on body fat and cardiometabolic risk factors in older (sarcopenic) obese people has not yet been clearly determined. However, there is significant evidence that high-protein diets generate favorable effects on parameters closely related to obesity parameters. This includes specific effects on appetite, hunger and satiety hormones [51, 52], significant enhancement of fat oxidation and increased thermogenesis [53, 54]. Further, isolated WPS augments muscle mass under isoenergetic conditions [21], preserves muscle mass under energy deficiency and induces more fat loss during energy restrictive diets in obese older adults [55, 56]. However, studies that assessed the effects of whey protein on fat reduction under isoenergetic conditions are rare and inconsistent. After 23 weeks of 56 g/d of WPS in overweight-obese adults, Baer et al. [13] reported a significant reduction of 2.3 kg in fat mass and 2.4 cm in waist circumference (WC) compared with an isoenergetic CHO supplementation. Tahavorgar et al. [57], who compared the effects of 12 weeks of whey versus soy protein preloads (65 or 60 g/d, 30 min before the largest meal of the day) in overweight to obese Persian males, 30–65 years old, described significant reductions of PBF and WC in both protein groups. However WPS resulted in much more pronounced reductions of PBF (− 9.1% vs. -3.1%), WC (− 9.7 vs. -2.3 cm) and appetite compared to soy protein. In contrast, after 12 weeks of intervention, Pal et al. [58] did not observe significant effects of WPS (54 g/d) on fat mass and distribution compared with an isoenergetic control group in a comparable cohort of overweight to obese adults. With respect to other markers of cardiometabolic health, a recent review [59] summarized predominately favorable effects on total cholesterol, LDL-C, HDL-C and TAG after 4–12 weeks of WPS using different doses. Likewise, the authors [59] reported positive effects of WPS on glucose control, inflammation/oxidative stress and blood pressure, however, the present literature summarized in this review is less consistent.

It was not our intention to address the aspect of whether protein supplementation increases the effect of WB-EMS on body composition, physical functioning and cardiometabolic indices. Rather, we aimed to determine (a) whether a recognized WB-EMS application [16, 21–27, 44] combined with a moderate to high dosed WPS intervention may positively impact cardiometabolic indices including body fat in men with SO, and (b) whether the (very) low threshold intervention “isolated WPS” may be sufficient to realize this aim.

Not perfectly addressed within the present research design, but based on the rather consistent effects of WB-EMS [16, 20–27, 44] on body composition provided by the literature, we think it is legitimate to discuss the effect of an additional WPS on recognized WB-EMS protocols with respect to body fat and cardiometabolic indices in older adults. The only study that focuses on people with SO, however, did not observe significant corresponding differences between WB-EMS with and without protein supplementation ([22, 44]. Enlarging the research area to the related RT literature, there is again limited evidence for the superiority of combined RT&Protein protocols (vs. isolated RT) on cardiometabolic risk and body fat indices. Several studies (e.g. [46–50, 60–65]) focus on this issue in middle-aged to older adults; however, based on a predominately positive effect on body fat and cardiometabolic risk factors in both groups [46, 48–50, 61–65], we were unable to locate any study that determined significant differences between the conditions. Of importance, however, studies vary considerably as to the protein source (whey/casein supplements, milk/red meat or combined egg/meat/milk diets) and dose (0.17 to 1.00 g/kg body mass/d) and frequently feature an insufficient sample size. Conversely, a recent study [65] with sufficient power that applied most of the recommendations given for optimum protein supplementation [7, 8, 66] did not report differences for body fat changes or cardiometabolic indices for their overweight to obese cohort 35–65 years old.

On the other hand, it is debatable whether the similar or predominately non-significantly more favorable results of the combined group compared to the easy feasible and very low threshold “protein-only” approach justify an additional WB-EMS (or RT) application; at least under the premise of significant effects on muscle mass in both treatment groups [21]. However, comparable to the reduction of fat parameters, the hypertrophic response of the WB-EMS&P was about twice as high^6^ as among the WPS group. Even more relevant for frail cohorts,^7^ functional parameters (i.e. grip-strength, gait speed) were not relevantly affected by the protein supplementation, while the combined protocol caused highly significant and clinically relevant effects. Thus, we strongly recommend a combined resistance type and protein intervention. Comparing WB-EMS with other resistance type exercises, its joint-friendly, time effective and highly customized mode of application predestines WB-EMS as a good option for older people either unable or unwilling to exercise conventionally.

Some limitations and features may decrease the scientific evidence of the FranSO study and our corresponding recommendations. (a) In contrast to present definitions [29, 67–69], subjects diagnosed as sarcopenic fulfilled only the morphological aspect of Sarcopenia (i.e. SMI < 0.789 kg/(kg/m^2^). (b) Following Baumgartner et al. [30], we selected a cut-off point of > 27% PBF for obesity. However, the average PBF in 2986 Caucasian males of Swiss origin 74–98 years old was reported to be 25.4 ± 5.1% (MV ± SD), corresponding to a BMI of 25.2 ± 3.0 kg/m^2^ [70]. Further, applying a T-Score based criterion (i.e. PBF-MV + 2 SD), based on our Northern Bavarian database of 1189 Caucasian men 18–35 years old [28], the corresponding cut-off point ranged around 31% PBF (24.5% for MV-1SD). (c) An additional isolated WB-EMS training group might have provided deeper insights. However, in fact, cdw men with sarcopenic obesity are rare [28]. Even though we weakened our inclusion criteria (see above), we are unable to generate another subgroup with sufficient statistical power. Since we determined the effects of an isolated WB-EMS group in a previous trial [22, 44], we opted to focus on the effect of isolated WPS on musculoskeletal and cardiometabolic risk factors in this study. (d) We used DSM-BIA to determine body composition. There are still some general concerns about this technology, at least when applied on adults with severe obesity [71] and when summarizing the results of different devices and equipment. We determined excellent agreements for PBF (Intra Class Correlation for PBF: 0.86 to 0.92 with consistently narrow limits of agreement) for lean-overweight cohorts of different ages between the DSM-BIA InBody 770 (Korea Seoul) used in this study and our DXA scanner (Hologic 4500a, Boston, USA), however. This observation was confirmed by Ling et al. [72] for the previous DSM-BIA version InBody 720 (Seoul, Korea) and an identical DXA scanner. (e) Due to unavailable validation data for the InBody 770, we considered “total visceral fat area”, as determined by DSM-BIA as an experimental endpoint. Nonetheless, using a small sample of the FranSO cohort demonstrated a good agreement between Magnet Resonance Imaging (MAGNETOM Skyra^fit^, Siemens Healthcare GmbH, Erlangen, Germany) and DSM-BIA (InBody 770; Seoul, Korea). However, this finding has to be confirmed with an adequate sample size. (f) We did not focus on “isoenergetic conditions” and hence did not supply the CG with a similar amount of non-protein derived energy (e.g. 50–60 g/d CHO for the CG in compensation for 51 and 59 g/d protein-supplementation in the treatment groups). Due to the specific assignment of protein for anabolic processes, the impact on thermoregulation, hunger and satiety along with its minor impact on energy metabolism during energy balance [73] and decreased energy efficiency [74], we think a corresponding approach might produce a more pronounced bias compared with an only theoretical computed difference for energy uptake. Of importance however, even when considering the 200–250 “extra kcal” of the protein supplementation, we did not detect a significant increase of energy uptake in the treatment groups (< 90 kcal/d). We attribute this finding to the frequently reported whey protein effects on appetite, hunger and satiety [51, 52, 57]. (g) The WB-EMS setting of the present study can be considered as personal training with one instructor and two users. This approach is also applied in the majority of commercial settings and ensures a high level of safety and effectivity [75]. However, a relevant part of the WB-EMS results can be contributed to the close interaction and may be independent of the training tool.

The generalization of our results to other cohorts may be problematic. Considering factors related to SO (e.g. inflammation [5], mitochondrial abnormalities [76], oxidative stress [77]) muscle response to exercise and/or protein may differ from healthy older people. On the other hand, WB-EMS trials with other cohorts but similar WB-EMS application demonstrated comparable results on obesity and cardiometabolic parameters [16, 23].

## Conclusion

FranSO is the first study to demonstrate the positive effect of whey protein supplementation, and to a more favorable extent, WB-EMS&Protein on obesity and cardiometabolic risk in cwd people 70+ with SO. The importance of this finding is obvious: Even in older men within the range of recent protein recommendations [7], higher whey protein doses not only affected muscle mass [21] but also reduced obesity and cardiometabolic indices. Adding exercise to this easy feasible approach significantly increases the effect on obesity (but not cardiometabolic) parameters. In this context, the joint-friendly, time effective and highly customized and personalized WB-EMS training technology that mitigate barriers and concerns about conventional resistance exercise may be a reasonable and safe option for older, functionally limited and frail people to fight obesity and sarcopenia.

## Abbreviations

ASMM: Appendicular skeletal muscle mass
cdw: Community dwelling
CG: Control group
CI: Confidence interval
DSM-BIA: Direct-segmental, multi-frequency Bio-Impedance Analysis
FNIH: Foundation for the National Institutes of Health
FORMOsA: “Forschungsverbund Muskelschwund (Sarkopenie) und Osteoporose – Folgen eingeschränkter Regeneration im Alter”
FranSO: Franconian sarcopenic obesity (study)
HDL-C: High density lipoprotein cholesterol
ITT: Intention to treat
LDL-C: Low density lipoprotein cholesterol
LLFDI: Late life function disability instrument
MV: Mean value
n.s.: Non-significant
PBF: Percent body fat
RPE: Rate of perceived exertion
RT: Resistance exercise training
SD: Standard deviation
SMI: Skeletal muscle mass index
SO: Sarcopenic obesity
TBF: Total body fat mass
TC/HDL-C: Total-cholesterol/HDL-cholesterol ratio
TF: Trunk fat mass
WB-EMS: Whole-body electromyostimulation
WB-EMS&P: Combined whole-body electromyo-stimulation and protein supplement group
WC: Waist circumference
WK: Wolfgang Kemmler
WPS: whey protein supplementation

## Footnotes

1 Ethikkommission der Friedrich-Alexander-Universität Erlangen-Nürnberg, Krankenhausstraße 12, 91,054 Erlangen, Germany; phone + 49 9131 85–22,270; e-mail: ethikkommission@fau.de

2 Appendicular skeletal muscle mass (ASMM)/ body mass index (BMI)

3 E.g. WB-EMS training every Monday/Tuesday and every second Thursday/Friday

4 Briefly, the chemical score indicates the “quality” of the protein source. The chemical score is calculated from the content of the amino acid with the lowest occurrence in the protein to be tested.The reference used is egg protein (100%).

5 Primary endpoint: Changes of Sarcopenia Z-Score [22] from baseline to 16 week follow-up

6 However, differences between WB-EMS&P versus WPS were also not significant (*p* = 0.055) for SMI

7 However, we did not consider our cdw cohort as “frail”
